# Changes in appetite, energy intake, body composition, and circulating ghrelin constituents during an incremental trekking ascent to high altitude

**DOI:** 10.1007/s00421-017-3683-0

**Published:** 2017-07-24

**Authors:** Jamie Matu, John O’Hara, Neil Hill, Sarah Clarke, Christopher Boos, Caroline Newman, David Holdsworth, Theocharis Ispoglou, Lauren Duckworth, David Woods, Adrian Mellor, Kevin Deighton

**Affiliations:** 1 Institute for Sport Physical Activity and Leisure, Leeds Beckett University, Leeds, LS6 3QS UK; 2 Section of Investigative Medicine, Imperial College London, London, UK; 3 Poole Hospital NHS Trust, Longfleet Rd, Poole, UK; 4 Royal Centre for Defence Medicine, ICT Building, Vincent Drive, Birmingham, UK

**Keywords:** Ghrelin, Hypoxia, Altitude-induced anorexia, Terrestrial altitude

## Abstract

**Purpose:**

Circulating acylated ghrelin concentrations are associated with altitude-induced anorexia in laboratory environments, but have never been measured at terrestrial altitude. This study examined time course changes in appetite, energy intake, body composition, and ghrelin constituents during a high-altitude trek.

**Methods:**

Twelve participants [age: 28(4) years, BMI 23.0(2.1) kg m^−2^] completed a 14-day trek in the Himalayas. Energy intake, appetite perceptions, body composition, and circulating acylated, des-acylated, and total ghrelin concentrations were assessed at baseline (113 m, 12 days prior to departure) and at three fixed research camps during the trek (3619 m, day 7; 4600 m, day 10; 5140 m, day 12).

**Results:**

Relative to baseline, energy intake was lower at 3619 m (*P* = 0.038) and 5140 m (*P* = 0.016) and tended to be lower at 4600 m (*P* = 0.056). Appetite perceptions were lower at 5140 m (*P* = 0.027) compared with baseline. Acylated ghrelin concentrations were lower at 3619 m (*P* = 0.046) and 4600 m (*P* = 0.038), and tended to be lower at 5140 m (*P* = 0.070), compared with baseline. Des-acylated ghrelin concentrations did not significantly change during the trek (*P* = 0.177). Total ghrelin concentrations decreased from baseline to 4600 m (*P* = 0.045). Skinfold thickness was lower at all points during the trek compared with baseline (*P* ≤ 0.001) and calf girth decreased incrementally during the trek (*P* = 0.010).

**Conclusions:**

Changes in plasma acylated and total ghrelin concentrations may contribute to the suppression of appetite and energy intake at altitude, but differences in the time course of these responses suggest that additional factors are also involved. Interventions are required to maintain appetite and energy balance during trekking at terrestrial altitudes.

## Introduction

Acute exposure to hypoxic environments has been demonstrated to suppress appetite and energy intake (Armellini et al. 1997; Matu et al. 2017; Wasse et al. 2012; Westerterp et al. 2000). This effect appears to be maintained during prolonged sojourns to high altitude, which results in significant decreases in body mass, of which greater than 50% is from fat-free mass (Rose et al. 1988; Sergi et al. 2010). These declines in lean mass will likely lead to a drop in physical capabilities at altitude (Sergi et al. 2010), which can have deleterious implications for individuals ascending to high altitude. A better understanding of the time course of these changes during a trek, as well as the mechanisms involved, is required to develop guidance for those travelling to high altitudes.

Over the past 20 years, changes in the circulating concentrations of gastrointestinal hormones and leptin have been implicated as potential mechanisms for the alterations in appetite and energy intake at altitude. However, although several hormones such as pancreatic polypeptide (Riepl et al. 2012), leptin (Sierra-Johnson et al. 2008), glucagon-like-peptide-1 (Snyder et al. 2008), and total ghrelin (Benso et al. 2007; Riepl et al. 2012; Shukla et al. 2005) have been measured in response to terrestrial altitude exposure, the findings remain equivocal. One major limitation of the current research is the measurement of total ghrelin concentrations at altitude, rather than the constituent components of acylated and des-acylated ghrelin which have opposing effects on appetite regulation (Fernandez et al. 2016). The differentiation of ghrelin constituents in response to terrestrial altitude is imperative as acylated ghrelin has been found to be particularly responsive to hypoxic exposure in a laboratory environment with decreases in this hormone correlated with a reduction in appetite (Bailey et al. 2015) and energy intake (Wasse et al. 2012). Furthermore, recent evidence suggests that des-acylated ghrelin may inhibit the orexigenic effects of acylated ghrelin (Fernandez et al. 2016), which further emphasises the need to measure both hormones as well as the ratio between the two (Al Massadi et al. 2014). It seems feasible that the measurement of total ghrelin in previous research (Benso et al. 2007; Debevec et al. 2014; Riepl et al. 2012) may have masked changes in acylated and des-acylated ghrelin, which may explain the lack of association between changes in appetite and circulating ghrelin concentrations at altitude.

Although circulating total ghrelin concentrations have been extensively investigated in response to hypoxic exposure (Benso et al. 2007; Debevec et al. 2014, 2016; Mekjavic et al. 2016; Riepl et al. 2012; Shukla et al. 2005), the investigation of acylated ghrelin is currently limited to four studies, all of which lasted for ≤7 h and were all conducted in normobaric environments (Bailey et al. 2015; Matu et al. 2017; Morishima and Goto 2016; Wasse et al. 2012). Although laboratory studies of this nature are valuable to gain greater mechanistic understanding, further field studies are required to assess the combined effects of trekking, gradual ascent, and other environmental stimuli such as cold exposure which occur during real life ascent to high altitude. The measurement of acylated and des-acylated ghrelin during ascent to terrestrial altitude is vital to understand the changes that occur during a real-world environment and the importance of these changes as a basis for the development of future interventions. The lack of investigation into the constituents of total ghrelin to date is likely due to the complexities of the necessary chemical preparation required to prevent the degradation of the analytes (Hosoda et al. 2004), which is particularly difficult to achieve in an extreme field environment.

The purpose of this study was to investigate the effects of a high-altitude trek to 5300 m on appetite, energy intake, and body composition responses in healthy men and women, with a further focus on circulating acylated and des-acylated ghrelin concentrations as mechanistic variables. These data provide novel insights into the time course of changes in appetite, energy intake, and body composition during a real-life ascent to high altitude. This study also provides a better understanding of the mechanisms responsible for altitude-induced anorexia, representing the first investigation of acylated ghrelin and des-acylated ghrelin at terrestrial altitude. We hypothesised that exposure to increasingly high altitudes would suppress appetite, circulating acylated ghrelin concentrations and energy intake, which would be associated with a reduction in lean and total body mass.

## Methods

### Participants

This study was conducted according to the guidelines laid down in the Declaration of Helsinki and all procedures were approved by the Ethics Advisory Committee at Leeds Beckett University and the Ministry of Defence Research Ethics Committee (MoDREC; protocol number 624). Twelve members (nine male and three female) of the British Military volunteered to participate in this study. Informed consent was obtained from all participants included in the study. All participants were non-smokers, had no known disease, allergies, or intolerances, and had not been to an altitude over 1000 m for at least 3 months. All participants were physically fit and could run 2.4 km on a treadmill at a 2% gradient in under 13 min 37 s in accordance with military requirements. The physical characteristics of participants [mean (SD)] were as follows: age 28 (4) years, body mass 71.3 (10.3) kg, and body mass index (BMI) 23.0 (2.1) kg m^−2^.

### Study design

This study represents part of the ‘British Services Dhaulagiri Medical Research Expedition’ which took place in March–May 2016 (Mellor et al. 2017). In April 2016, participants in the present study travelled from the UK to Nepal and completed a 14-day trek around the Dhaulagiri circuit in the Himalayas. Travel from the UK to Nepal lasted for 1 day and participants were in Nepal for 3 days prior to starting the trek. The trek commenced from Darbang (~1100 m), peaked on day 11 at the French Pass (~5300 m), and ended on day 14 at Marpha (~2700 m). Pre-planned rest days were included at fixed camps at 3619 m (Camp 1; day 7), 4600 m (Camp 2; day 10), and 5140 m (Camp 3; day 12). Participants walked a mean distance of 8.2 km day^−1^ with a mean elevation gain of 471 m day^−1^ whilst carrying a day pack weighing ~5 kg. Further information about the ascent profile and trek characteristics has been published elsewhere (Mellor et al. 2017). Data collection took place at baseline (113 m; 12 days prior to departure from the UK) and at each fixed camp. On the day preceding data collection at camp 1, camp 2, and camp 3 participants walked 4.3, 4.3, and 9.1 km and gained an elevation of 512, 528, and 540 m, respectively. All trekking on these days was completed by 5 pm. All participants wore the same type of clothing throughout the trek and experienced the same degree of cold exposure. Baseline measurements were collected in the laboratories at Leeds Beckett University and the measures at each camp were collected in a designated research tent. At the time data were collected, ambient temperatures in the laboratory and research tents were 19.8, 4.9, 1.2, and −6.4 °C at baseline, 3619, 4600, and 5140 m, respectively. All participants remained rested on the day of testing. Participants were staggered for the collection of all measurements between 7 am and 10 am, with each participant having their measures taken at a consistent time on all occasions and after an overnight fast of at least 10 h.

### Food provision

Throughout the trek, all foods and fluids were available ad libitum, and were provided by Nepalese cooks and staff who accompanied the trekking team. Typical foods offered at each meal were as follows: breakfast—cereals, porridge, omelette, and pancakes; lunch—noodles, meats, soup, beans, vegetables, and fruit; dinner—curry, pasta, pizza, potatoes, dumplings, cheese, and vegetables; and snacks—chocolate bars, biscuits, cake, and fruit. At all meals, participants were given more than one option and thus could decide what they wanted to eat. The mean (SD) macronutrient composition of the food consumed during the trek was 49.0 (6.6) % carbohydrate, 36.3 (6.2) % fat, and 14.7 (2.6) % protein, respectively.

### Food intake

Energy intake was assessed at baseline via a 24-h dietary recall interview by an experienced researcher (Academic Associate of the Sport and Exercise Nutrition Register) using the multiple-pass approach (Guenther et al. 1997). In addition to collecting dietary intake information, this approach was used to demonstrate the level of detail required from the participants when completing a food diary during the trek. All participants completed a food diary on the day preceding each fixed camp and this process was monitored and verified by the same experienced researcher throughout the trek. Although there are acknowledged limitations of self-reported dietary intake methods (Hill and Davies 2001), the oversight of food diaries by the researcher present on the trek ensured accurate completion of all food diaries. Food intake was monitored during the day before each fixed camp to consider acute dietary changes when interpreting the data for fasted appetite ratings and blood samples at the fixed camps. The palatability of each meal consumed was measured using 100 mm visual analogue scales (VAS) with the anchors “the worst taste that I have ever experienced” and “the best taste that I have ever experienced” at each end of the scale. A mean palatability score was calculated for each day to control for any influence of palatability on food consumption.

### Appetite

Appetite perceptions were measured after an overnight fast at baseline and upon waking at each fixed camp using validated 100 mm VAS for hunger, satisfaction, fullness, and prospective food consumption (PFC) (Flint et al. 2000). Using these scales, a composite appetite score (CAS) was calculated using the following formula: composite appetite score = ([hunger + prospective food consumption + (100−fullness) + (100−satisfaction)]/4) (Stubbs et al. 2000). A higher value is associated with a greater appetite sensation and subsequently a stronger motivation to eat. In addition, the extent to which participants desired sweet, salty, savoury, and fatty foods was assessed using VAS anchored at each end with “yes, very much”, and “no, not at all”.

### Acute mountain sickness, oxygen saturation, and rating of perceived exertion

Acute mountain sickness (AMS) was assessed every morning and evening using the Lake Louise AMS (LLS) score (Roach et al. 1993); mild AMS was defined as LLS of ≥3 in the presence of a headache and severe AMS was defined as LLS of ≥6 in the presence of a headache. Arterial oxygen saturations (SpO_2_) were measured via a fingertip pulse oximeter (Nellcor™ PM10N; Medtronic, Minneapolis, MN) every morning and evening, whilst the participants were resting in a seated position. Rating of perceived exertion (RPE) (Borg 1982) was recorded as the hardest exertion experienced during the day of trekking preceding each fixed camp, as used previously in similar environments (Mellor et al. 2014).

### Body composition

Participants were weighed at baseline and each fixed camp in a fasted state whilst wearing minimal clothing and no footwear. A portable multicomponent force plate (Kistler, Switzerland) was used and was stabilised on the mountain using specialised levelling feet (JVD Design & Automation Ltd, Leeds, UK). Skinfolds of the triceps, subscapular, biceps, iliac crest, supraspinale, abdominal, front thigh, and medial calf were measured using calibrated Harpenden callipers (John Bull, British Indicators, West Sussex, UK) to the nearest 0.1 mm. The sum of skinfolds was calculated by the addition of each of the eight skinfold values in mm. Girth measurements of the waist and calf, as well as the upper arm in a relaxed and flexed state, were obtained using a steel anthropometric tape (Lufkin W606PM, Cooper Hand Tools, Tyne & Wear, UK) to the nearest 1 mm. All anthropometric assessments were conducted by one researcher who was trained by an individual accredited by the International Society for the Advancement of Kinanthropometry (ISAK). All measures were conducted in duplicate and in accordance with ISAK guidelines on the right side of the body. The coefficient of variation for skinfolds and girths was 2.2 and 0.4%, respectively.

### Blood sampling

Venous blood samples were obtained from an antecubital vein via venepuncture using a 21-gauge butterfly needle (Safety-Lok™; BD, Oxford, UK). Samples were collected at baseline and at all research camps with participants in a fasted state. One 4.9 mL pre-cooled EDTA monovette (Sarstedt, Leicester, UK) was used to obtain samples for the determination of plasma acylated and des-acylated ghrelin concentrations. Monovettes were pre-treated on the morning of testing, to prevent the degradation of acylated ghrelin, with 50 µL of a solution containing p-hydroxymercuribenzoic acid, potassium phosphate buffer, and sodium hydroxide (Hosoda et al. 2004). Immediately after filling, the tube was spun at 1500×*g* for 10 min in a centrifuge (CompactStar CS4, VWR). Subsequently, 1 mL of plasma was mixed with 100 µL of 1 M hydrochloric acid. This solution was then immediately frozen at either −20 °C in a freezer (for baseline measurements) or within a dry shipper containing liquid nitrogen at <−80 °C (at each fixed camp) before being transferred to a −80 °C freezer at the university and stored until analysis. Plasma volume changes as a result of altitude exposure were not assessed during the trek, because it is the absolute plasma hormonal concentrations that would determine the body’s response at that specific time (Kargotich et al. 1998). However, to prevent any extraneous influences from postural changes, all blood samples were collected after the participant had been seated for at least 5 min (Fawcett and Wynn 1960).

### Blood analyses

Commercially available enzyme immunoassays were used to determine plasma concentrations of acylated and des-acylated ghrelin (SPI BIO, Montigny Le Bretonneux, France). To eliminate interassay variation, all samples from each participant were analysed on the same plate. In addition, all samples were analysed in duplicate and on the same day. The within batch coefficients of variation were 2.5% for acylated ghrelin and 2.4% for des-acylated ghrelin. Total ghrelin was computed via the addition of acylated and des-acylated ghrelin concentrations. The ratio between acylated ghrelin and des-acylated ghrelin concentrations (AG:DG) was calculated as acylated ghrelin divided by des-acylated ghrelin, as previously described (Delhanty et al. 2015).

### Statistical analysis

Data are expressed as mean (SD) in text and tables and mean (SE) in figures to avoid distortion of the graphs. Diet records were inputted into the Nutritics dietary analysis software (v1.8 for Windows; Nutritics, Dublin) to assess energy intake. All data were analysed using IBM SPSS statistics (v22.0 for Windows; SPSS, Chicago, IL). One-way repeated measures analysis of variance (ANOVA) was used to assess altitude-based differences in SpO_2_, AMS scores, RPE scores, body composition measures, appetite perceptions, energy intake, fluid intake, and plasma ghrelin concentrations. Significant effects were further explored using Student’s paired *t* tests. Effect sizes are presented as Cohen’s *d* and interpreted as ≤0.2 trivial, >0.2 small, >0.6 moderate, >1.2 large, >2 very large, and >4 extremely large (Hopkins 2004). The Pearson product moment correlation coefficient was used to investigate relationships between variables at each altitude. The exclusion of participants reporting AMS did not alter the interpretation of the findings; subsequently, all participants were included in the data analysis. Based on evidence that males and females exhibit similar appetite, energy intake, and gut hormone responses to exercise- and diet-induced energy deficits (Alajmi et al. 2016), data from both genders were combined for analyses. The sample size used within this study was deemed sufficient to detect a significant difference in energy intake between altitudes. The anticipated effect size for a difference in energy intake was based on a similar previous study which investigated energy intake in individuals climbing at approximately 4500 m for 16 days (Armellini et al. 1997). Based on the effect size and an alpha value of 5%, a sample size of 12 participants would generate a power >95%. Calculations were performed using G*power (v3 for Windows; Düsseldorf) (Faul et al. 2007).

## Results

Measurements of SpO_2_, AMS, RPE, body composition, fluid intake, and energy intake were successfully obtained from all 12 participants. One male participant withdrew consent for blood sampling at the research camps during the trek and one male participant did not complete the appetite perception measurements during the trek. Therefore, data are presented for 11 participants for plasma hormone concentrations and appetite perceptions.

### Oxygen saturations, acute mountain sickness, and rating of perceived exertion

One-way ANOVA revealed a main effect of altitude for SpO_2_ (*P* < 0.001). Post-hoc analysis demonstrated lower SpO_2_ at each fixed camp compared with the previous location [baseline: 98.4 (0.9) %; 3619 m: 92.4 (2.5) %, *P* < 0.001, *d* = 3.19; 4600 m: 83.5 (4.1) %, *P* < 0.001, *d* = 2.62; 5140 m: 79.8 (5.6) %, *P* = 0.007, *d* = 0.75]. A positive diagnosis of mild AMS was reported in 50% of participants at some point during the trek. The first incidence of AMS occurred on the 8th day of the trek (the day after the first rest day; 4072 m). Incidence of AMS at the four fixed locations was as follows: baseline: zero participants; 3619 m: zero participants; 4600 m: two participants; and 5140 m: two participants. One-way ANOVA revealed a significant effect of altitude for RPE (*P* < 0.001). Relative to 3619 m [11.8 (1.5)], RPE was significantly higher at 4600 m [13.3 (1.5), *P* = 0.009, *d* = 1.01] and relative to 4600 m RPE was significantly higher at 5140 m [16.5 (2.5), *P* = 0.003, *d* = 1.58].

### Energy and fluid intake

**Fig. 1:**
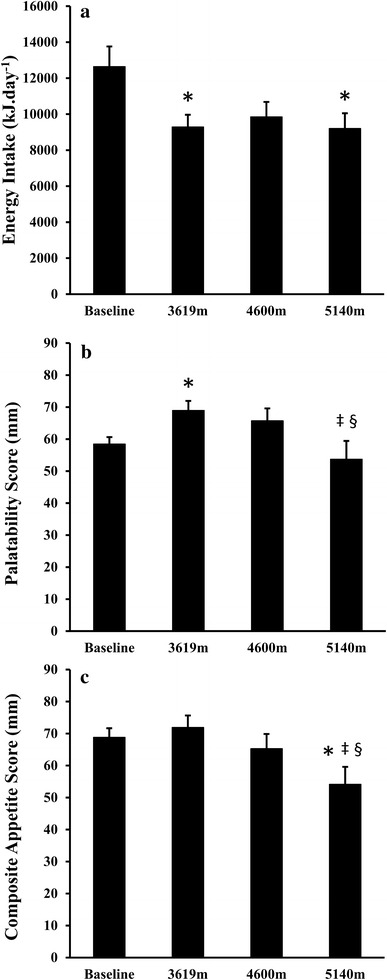
Energy intake (**a**), palatability score (**b**) and composite appetite score (**c**) at baseline, 3619, 4600, and 5140 m. * significant difference from baseline. ‡ significant difference between 4600 and 5140 m. § significant difference between 3619 and 5140 m (one-way ANOVA; *P* < 0.05 after post-hoc analyses). Values are mean (SE), *N* = 12 for energy intake and palatability, *N* = 11 for composite appetite score

One-way ANOVA revealed a significant effect of altitude for energy intake (*P* = 0.015). Relative to baseline, energy intake was significantly lower at 3619 m (*P* = 0.038, *d* = 1.05) and 5140 m (*P* = 0.016, *d* = 1.00) and tended to be lower at 4600 m (*P* = 0.056, *d* = 0.82). There were no differences observed between research camps during the trek (all *P* ≥ 0.333, *d* ≤ 0.22) (Fig. 1a).

One-way ANOVA revealed a main effect of altitude for fluid intake (*P* = 0.029). Relative to baseline (2769 (1156) mL day^−1^), fluid intake was significantly higher at 3619 m [4438 (1847) mL day^−1^, *P* = 0.008, *d* = 1.08] and 4600 m [4236 (2120) mL day^−1^, *P* = 0.027, *d* = 0.86], but not significantly higher at 5140 m [3645 (2026) mL day^−1^, *P* = 0.126, *d* = 0.53]. There were no differences observed between camps (all *P* ≥ 0.266, *d* ≤ 0.29).

One-way ANOVA revealed a main effect of altitude for the daily palatability of food consumed (*P* = 0.018). Relative to baseline, palatability was significantly higher at 3619 m (*P* = 0.030, *d* = 1.17). However, palatability was not different at 4600 m (*P* = 0.147, *d* = 0.66) or 5140 m (*P* = 0.509, *d* = 0.32) compared with baseline. Palatability was significantly lower at 5140 m compared with 3619 m (*P* = 0.020, *d* = 0.97) and 4600 m (*P* = 0.013, *d* = 0.71) (Fig. 1b).

One-way ANOVA revealed a main effect of altitude on the desire to eat salty (*P* = 0.025) and savoury (*P* < 0.001) foods, but not sweet (*P* = 0.604) or fatty (*P* = 0.354) foods. Relative to baseline [67 (12) mm], the desire to eat salty foods was significantly lower at 3619 m [41 (23) mm, *P* = 0.018, *d* = 1.42] and 5140 m [39 (27) mm, *P* = 0.024, *d* = 1.38] and also tended to be lower at 4600 m [45 (27) mm, *P* = 0.066, *d* = 1.09]. There were no differences observed between camps (all *P* ≥ 0.159, *d* ≤ 0.22). The desire to eat savoury foods was significantly increased at 3619 m [70 (13) mm, *P* < 0.001, *d* = 2.41], 4600 m [67 (15) mm, *P* < 0.001, *d* = 2.03], and 5140 m [53 (19) mm, *P* = 0.011, *d* = 1.08) compared with baseline [33 (18) mm]. In addition, the desire to eat savoury foods reduced significantly from 4600 to 5140 m (*P* = 0.026, *d* = 0.79) with no difference observed between 3619 and 4600 m (*P* = 0.516, *d* = 0.23).

### Appetite perceptions

One-way ANOVA revealed a main effect of altitude for CAS (*P* = 0.005). Post-hoc analysis revealed that CAS was significantly lower at 5140 m compared with baseline (*P* = 0.027, *d* = 1.07), 3619 m (*P* = 0.005, *d* = 1.19), and 4600 m (*P* = 0.05, *d* = 0.69). No other differences were observed between altitudes (all *P* ≥ 0.116, *d* ≤ 0.48) (Fig. 1c).

### Plasma acylated and des-acylated ghrelin concentrations

**Fig. 2:**
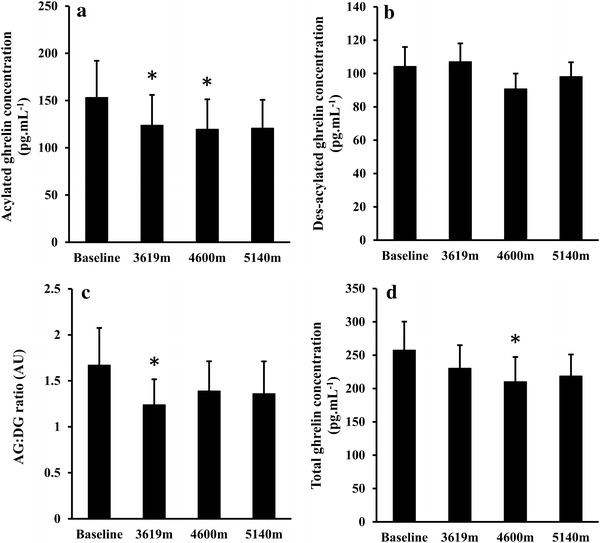
Acylated ghrelin (**a**), des-acylated ghrelin (**b**), AG:DG ratio (**c**), and total ghrelin (**d**) concentrations at baseline, 3619, 4600, and 5140 m. * significant difference from baseline (one-way ANOVA; *P* < 0.05 after post-hoc analyses). Values are mean (SE), *N* = 11

One-way ANOVA revealed a significant effect of altitude for plasma acylated ghrelin concentrations (*P* = 0.048; Fig. 2a), plasma total ghrelin concentrations (*P* = 0.047; Fig. 2d), and the AG:DG ratio (*P* = 0.046; Fig. 2c). A main effect of altitude was not detected for plasma des-acylated ghrelin concentrations (*P* = 0.177; Fig. 2b).

Relative to baseline, plasma acylated ghrelin concentrations were significantly lower at 3619 m (*P* = 0.046, *d* = 0.25) and 4600 m (*P* = 0.038, *d* = 0.29), and tended to be lower at 5140 m (*P* = 0.070, *d* = 0.28). There were no differences observed between camps (all *P* ≥ 0.512, *d* ≤ 0.04; Fig. 2a). Plasma AG:DG ratio decreased significantly from baseline to 3619 m (*P* = 0.034, *d* = 0.37), and tended to be lower than baseline at 4600 m (*P* = 0.069, *d* = 0.23) and 5140 m (*P* = 0.070, *d* = 0.25). There were no differences observed between camps (all *P* ≥ 0.362, *d* ≤ 0.15) (Fig. 2c). Plasma total ghrelin concentrations decreased significantly from baseline to 4600 m (*P* = 0.045, *d* = 0.36); however, no other significant differences were observed between altitudes (all *P* ≥ 0.111, *d* ≤ 0.31) (Fig. 2d).

### Body composition

One-way ANOVA revealed a significant effect of altitude for body mass (*P* < 0.001), sum of skinfolds (*P* < 0.001), calf girth (*P* = 0.010), waist girth (*P* = 0.016), and relaxed arm girth (*P* = 0.029), with no significant differences observed for flexed arm girth (*P* = 0.173) (Table 1).

**Table 1:** Body composition measurements at baseline, 3619, 4600, and 5140 m

	Baseline	3619 m	4600 m	5140 m
Body mass (kg)	71.3 (10.3)	73.1 (10.2)*	70.8 (10.7)^†^	71.1 (10.0)^§^
Sum of 8 Skinfolds (mm)	81.2 (23.7)	74.2 (22.3)*	73.5 (21.7)*	75.5 (23.9)*
Calf girth (cm)	38.1 (1.9)	37.5 (2.1)	37.2 (2.2)*^,†^	36.9 (2.1)*^,§^
Waist girth (cm)	77.5 (6.6)	78.2 (5.5)	77.3 (5.5)	76.6 (5.4)^‡,§^
Relaxed arm girth (cm)	29.5 (3.0)	28.6 (3.0)*	28.8 (3.1)*	28.9 (2.9)^§^
Flexed arm girth (cm)	30.6 (3.2)	30.2 (3.2)	30.1 (3.2)	30.1 (3.1)

Values are mean (SD), *N* = 12

* Significant difference from baseline

^†^ Significant difference between 3619 and 4600 m

^‡^ Significant difference between 4600 and 5140 m

^§^ Significant difference between 3619 and 5140 m (one-way ANOVA; *P* < 0.05 after post-hoc analyses)

Body mass increased from baseline to 3619 m (*P* = 0.002, *d* = 0.18), decreased between 3619 and 4600 m (*P* < 0.001, *d* = 0.22) and did not change between 4600 and 5140 m (*P* = 0.415, *d* = 0.03). Sum of skinfolds was lower at 3619 m (*P* = 0.001, *d* = 0.30), 4600 m (*P* < 0.001, *d* = 0.34), and 5140 m (*P* = 0.001, *d* = 0.24) compared with baseline. There were no significant differences observed between each of the camps during the trek (all *P* ≥ 0.116, *d* ≤ 0.09).

Calf girth did not differ significantly between baseline and 3619 m (*P* = 0.127, *d* = 0.30), however, was significantly decreased at 4600 m (*P* = 0.039, *d* = 0.44) and 5140 m (*P* = 0.008, *d* = 0.60) compared with baseline. Calf girth was also significantly lower at 4600 m compared with 3619 m (*P* = 0.031, *d* = 0.14), and tended to be lower at 5140 m compared with 4600 m (*P* = 0.069, *d* = 0.14). Waist girth did not differ between baseline and any of the three camps (all *P* ≥ 0.122, *d* ≤ 0.15), however, was significantly lower at 5140 m than 3619 m (*P* < 0.001, *d* = 0.29) and 4600 m (*P* = 0.04, *d* = 0.13). Relaxed arm girth was significantly lower at 3619 m (*P* = 0.022, *d* = 0.30) and 4600 m (*P* = 0.047, *d* = 0.23) and tended to be lower at 5140 m (*P* = 0.073, *d* = 0.20) compared with baseline. There was a significant increase in relaxed arm girth between 3619 and 5140 m (*P* = 0.047, *d* = 0.10), with no other differences observed between camps (all *P* ≥ 0.191, *d* ≤ 0.07).

### Correlations

There were no correlations observed at any altitude between energy intake and CAS (all *r* ≤ 0.311, *P* ≥ 0.352). At 3619 m, CAS tended to be associated with plasma acylated ghrelin (*r* = 0.603, *P* = 0.065) and total ghrelin (*r* = 0.626, *P* = 0.053) concentrations. In addition, at 3619 m, energy intake was significantly correlated with des-acylated ghrelin concentrations (*r* = 0.686, *P* = 0.029). At 4600 m, CAS was significantly correlated with acylated ghrelin concentrations (*r* = 0.633, *P* = 0.049) and the AG:DG ratio (*r* = 0.667, *P* = 0.035). There were no other significant correlations observed between any variable, at any altitude (all *r* ≤ 0.511, *P* ≥ 0.108).

## Discussion

This study presents an assessment of the changes in appetite perceptions, energy intake, body composition, and ghrelin constituents throughout a trek to high terrestrial altitude. The findings demonstrate a reduction in energy intake and skinfold thickness during the trek, with a progressive reduction in appetite at increasing altitudes. This study provides the first investigation of acylated ghrelin and des-acylated ghrelin concentrations at terrestrial altitude and demonstrates a suppression of acylated- but not des-acylated ghrelin during the trek. These findings highlight the importance of measuring ghrelin constituents in addition to total ghrelin concentrations as small fluctuations in des-acylated ghrelin may mask changes in acylated ghrelin if only total ghrelin was to be measured. This phenomenon would have occurred at 3619 m in the present study as observed by a significant decrease in acylated ghrelin levels in the absence of any significant changes in des-acylated and total ghrelin concentrations. The findings from this study also demonstrate the need for interventions to maintain appetite during exposure to terrestrial altitudes, particularly above 4600 m.

In the present study, energy intake was reduced by 27% (9326 kJ) at 3619 m, 22% (9886 kJ) at 4600 m, and 27% (9238 kJ) at 5140 m compared with baseline, which substantiates previous findings at similar altitudes. Armellini et al. (1997) observed a 29% decrease in energy intake in individuals climbing at approximately 4500 m for 16 days, whilst Aeberli et al. (2013) demonstrated a 32% reduction in energy intake 2 days after rapid ascent to 4559 m. One study, however, found that energy intake, as well as fat and muscle mass, could be maintained up to an altitude of 5050 m when a wide choice of palatable foods was available in a comfortable setting (Kayser et al. 1993). It may therefore be argued that the reduction in energy intake in the present study was caused by a lack of food availability and reduced palatability of food whilst trekking in a foreign country. However, at 3619 m, energy intake was significantly suppressed compared with baseline, whilst food was widely available and the mean palatability of the food consumed was significantly higher than baseline. These findings agree with those of Rose et al. (1988) who found a significant reduction in ad libitum energy intake during a simulated ascent of Mount Everest, despite a variety of palatable foods being available. Despite the reduction in energy intake, fasting appetite perceptions were similar between baseline and 3619 m, which suggest a greater satiating effect of the energy consumed. This response was maintained at 4600 m, but appetite perceptions decreased significantly at 5140 m despite consistent food intake. Observations during the trek suggested that participants were consciously trying to maintain energy intakes throughout the trek in an attempt to maintain physical performance. This would support the observation that food intake was similar between the three camps, but that appetite perceptions and the desire for foods decreased with increasing altitude. This mismatch between appetite perceptions and energy intake is further supported by the lack of correlation between the two variables at each altitude. The reduced palatability of the foods consumed at 5140 m also supports a reduction in appetite, and occurred despite the same ad libitum food provision throughout the trek. This effect accords with the findings from previous animal studies which suggest that hypoxia degrades the taste of food (Ettinger and Staddon 1982). Considering the significant suppression of appetite at 5140 m, it is unclear whether food intakes could continue to be maintained over a more prolonged period and targeted interventions to better maintain appetite above 4600 m may be beneficial.

From a mechanistic perspective, trekking to high altitude induced a suppression of acylated but not des-acylated ghrelin concentrations, which resulted in a suppression of total ghrelin levels and the AG:DG ratio. However, at 5140 m, the only altitude in which CAS was significantly suppressed, no correlations were observed between any blood marker and CAS or energy intake. These findings suggest that appetite regulation during high-altitude trekking may be influenced by other hormonal [e.g., leptin, glucagon-like peptide-1, and peptide YY (Debevec 2017)] and non-hormonal [e.g., taste degradation (Ettinger and Staddon 1982) potentially altering food reward (Berthoud 2006)] factors. Appetite regulation is a complex multifaceted system which involves the integration of a wide range of neuroendocrine and psychological factors (Murphy and Bloom 2006). Subsequently, appetite suppression at altitude is unlikely to be solely explained by the measurement of a single hormone. However, a better understanding of the neuroendocrine responses to high-altitude trekking could be beneficial in the design of interventions to minimise appetite suppression at altitude.

The reductions in fasted acylated and total ghrelin concentrations, the day after significantly reduced energy intakes compared with baseline measurements, are particularly interesting considering the evidence that ghrelin levels and appetite perceptions increase in response to reduced food intake at sea level (Alajmi et al. 2016). Furthermore, acylated ghrelin levels remained depressed during the trek despite reductions in body mass between 3619 and 4600 m and the established inverse relationship between body mass and ghrelin concentrations at sea level (Chen et al. 2009; Shiiya et al. 2002). These observations suggest that the reductions in acylated and total ghrelin during this study were genuine effects of high-altitude exposure rather than being secondary to any changes in food intake or body composition. Although these changes in ghrelin were small, they appear to be physiologically relevant as changes of this magnitude have previously been associated with reductions in appetite and energy intake in a laboratory environment (Bailey et al. 2015; Wasse et al. 2012). It would be beneficial for future research to attempt to increase circulating plasma acylated ghrelin concentrations at altitude, to quantify these effects on appetite responses. Potential methods of accomplishing this include ghrelin infusion (Druce et al. 2005) or dietary interventions to manipulate ghrelin constituents [e.g., increased medium chain triglyceride intake as a substrate for ghrelin acylation (Kawai et al. 2017; Nishi et al. 2005)].

The reasons for the observed suppression of acylated ghrelin at altitude are unclear. However, considering the lack of change in total ghrelin levels at 3619 m, it seems plausible that the post-translational acylation of ghrelin may have been inhibited during the early stages of the trek due to inhibited ghrelin-*O*-acyltransferase (GOAT) activity or reduced availability of medium chain fatty acids as the substrate for acylation (Nishi et al. 2005). Alternatively, the reduction in both acylated ghrelin and total ghrelin at 4600 m suggests inhibited secretion of ghrelin from the P/D1 cells of the stomach (Kojima et al. 1999). A reduction in gut blood flow at altitude (Loshbaugh et al. 2006) has also been proposed to reduce ghrelin concentrations (Wasse et al. 2012); however, this concept has been disputed (Kalson et al. 2010; Mekjavic et al. 2016). The depression of acylated ghrelin levels prior to the depression of total ghrelin levels at the subsequent research camp suggests that acylated ghrelin may be a more sensitive measure of altered appetite signalling at altitude. Furthermore, the AG:DG ratio of >1 in the present study supports recent data that acylated ghrelin constitutes a much larger proportion of total ghrelin than previously thought (Delhanty et al. 2015) and demonstrates that preservation of this peptide can be achieved during field research in extreme environments.

In accordance with previous research, considerable changes in body composition were observed during the trek. This includes a mean reduction in body mass of 2.3 kg in the 3 days between 3619 and 4600 m, which was associated with significant decreases in calf girth despite very high levels of physical activity (mean distance walked: 8.8 km day^−1^, mean elevation gain: 491 m day^−1^). It seems likely that the reductions in body mass were not only caused by decreases in energy intake, but also by increases in energy expenditure due to the high-altitude environment and high physical activity levels. Such decreases in body and muscle mass have been observed in previous altitude research (Benso et al. 2007; Rose et al. 1988; Shukla et al. 2005; Westerterp et al. 2000) and it is likely that these losses would impair physical performance in these environments (Sergi et al. 2010). The further reductions in calf girth between 3619 and 5140 m also align with previous research (Rose et al. 1988), but the reasons for this response are unknown. We speculate that an increase in protein degradation may have occurred due to increasing altitude exposure (Holm et al. 2010). However, contrary to these changes in calf girth, an increase in relaxed arm girth was observed between 3619 and 5140 m. Although this increase was statistically significant, the absolute increase of 0.3 mm seems trivial. This may represent a compensatory response to the significant reduction in arm girth between baseline and 3619 m which is most likely due to atrophy caused by reduced activity of the arms during final preparations and low altitude trekking. Despite these insights into changes in body composition, it must be acknowledged that the baseline measures were collected 12 days before departure from the UK, which may have confounded comparisons between baseline values and those obtained during the trek due to changes in body composition during the final preparations for the expedition. Potential increases in lean body mass from final preparations and reductions in body fat would support the observed increase in body mass at the first fixed camp and reduced skinfold values at all camps relative to baseline. At sea level, an increase in body mass would usually result in a reduction in acylated ghrelin concentrations (Chen et al. 2009), which accords with our findings at the first fixed camp. However, in the present study, body mass then significantly reduced at the second and third camps, without a subsequent increase in ghrelin. This suggests that changes in ghrelin were unlikely to be the result of fluctuations in body mass during the trek and were more strongly mediated by high-altitude exposure.

Although the findings of the present study provide novel information regarding the appetite and metabolic responses during an incremental trekking ascent to high altitude, some notable limitations must be acknowledged. First, the current study design did not include a control group to separate the effects of trekking and high-altitude exposure. Therefore, it is not possible to conclude that the obtained results are a consequence of hypobaric hypoxia per se, but are a result of high-altitude trekking which combines various environmental and psychological factors as well as demanding physical exercise. An example of this being that cold exposure may interfere with energy balance, potentially by increasing non-shivering thermogenesis (van der Lans et al. 2013). Although this limits interpretation of the influence of each of these factors individually, the study design allowed us to investigate the effects of a real-world gradual ascent to high altitude to understand the practical implications for high-altitude trekking. Second, it was not possible to standardise the trekking distance on the day preceding each fixed camp due to the extreme terrain and environment. The greater trekking distance performed on the day prior to the final research camp in combination with higher altitude exposure resulted in markedly higher RPE scores at 5140 m. However, although exercise-induced anorexia occurs acutely in response to strenuous exercise, tightly controlled laboratory studies suggest that this does not affect appetite perceptions and ghrelin concentrations during the next day (King et al. 2015). Therefore, the suppression of appetite at 5140 m is unlikely to be due to greater exertion during trekking on the previous day. Furthermore, increased energy expenditure from the greater trekking distance and the continued suppression of energy intake suggests that participants would have been in their greatest energy deficit at the final research camp. This would be expected to increase appetite under sea-level conditions (Alajmi et al. 2016) and provides further support for a genuine altitude-mediated suppression of appetite. Third, the current study did not assess hydration status during the trek; therefore, it is possible that the observed reduction in body mass could be partly attributed to dehydration. However, it should be noted that reductions in skinfold and girth measurements were observed during the trek which suggests that the changes in body mass were at least partly due to genuine reductions in fat mass and muscle mass. Although the estimation of body composition using skinfold and girth measurements contains limitations, this was deemed to be the most practical and achievable method of assessment considering the extreme research environment encountered in the present study. In addition, mean fluid intake on the day before each fixed camp was >3.6 L day^−1^ which makes it unlikely that the participants experienced severe dehydration. It is not expected that the higher fluid intake during the trek, compared with baseline, would have influenced the measured ghrelin constituents given that gastric distension from water ingestion does not appear to influence plasma ghrelin concentrations (Tschop et al. 2000).

In conclusion, this study represents the first investigation of circulating ghrelin constituents in response to terrestrial altitude exposure and provides a time course of the changes in appetite, energy intake and body composition during gradual trekking ascent to high altitude. These findings demonstrate consistently reduced energy intake during high-altitude exposure and an incremental reduction in appetite perceptions with increasing altitude. These changes were associated with reductions in circulating concentrations of acylated and total ghrelin during the trek but differences in the time course of these responses suggests that additional factors are also involved. A negative energy balance during the trek caused reductions in body mass and lower body muscle mass which may have negative consequences for physical performance. Future investigations are required to develop nutritional and/or physiological interventions to maintain appetite, energy intake, and muscle mass at altitude.

## Abbreviations

AG:DG: Acylated ghrelin to des-acylated ghrelin ratio
AMS: Acute mountain sickness
ANOVA: Analysis of variance
BMI: Body mass index
CAS: Composite appetite score
GOAT: Ghrelin-*O*-acyltransferase
ISAK: International Society for the Advancement of Kinanthropometry
LLS: Lake Louise Score
MoDREC: Ministry of Defence Research Ethics Committee
RPE: Rating of perceived exertion
SD: Standard deviation
SE: Standard error
SpO_2_: Arterial oxygen saturations
VAS: Visual analogue scales
